# Evaluation of the growth-inducing efficacy of various *Bacillus* species on the salt-stressed tomato (*Lycopersicon esculentum* Mill.)

**DOI:** 10.3389/fpls.2023.1168155

**Published:** 2023-03-28

**Authors:** Anil Patani, Dharmendra Prajapati, Daoud Ali, Haresh Kalasariya, Virendra Kumar Yadav, Jigna Tank, Snehal Bagatharia, Madhvi Joshi, Ashish Patel

**Affiliations:** ^1^ Department of Biotechnology, Smt. S. S. Patel Nootan Science and Commerce College, Sankalchand Patel University, Visnagar, Gujarat, India; ^2^ Department of Zoology, College of Science, King Saud University, Riyadh, Saudi Arabia; ^3^ Centre for Natural Products Discovery, School of Pharmacy and Biomolecular Sciences, Liverpool John Moores University, Liverpool, United Kingdom; ^4^ Department of Life Sciences, Hemchandracharya North Gujarat University, Patan, Gujarat, India; ^5^ Department of Biosciences, Saurashtra University, Rajkot, Gujarat, India; ^6^ Gujarat State Biotechnology Mission (GSBTM), Udyog Bhavan, Gandhinagar, Gujarat, India; ^7^ Gujarat Biotechnology Research Centre (GBRC), Gandhinagar, Gujarat, India

**Keywords:** PGPR, salt stress, tomato, plant growth promotion, antioxidant enzymes

## Abstract

Plants are affected by salt stress in a variety of ways, including water deficiency, ion toxicity, nutrient imbalance, and oxidative stress, all of which can cause cellular damage or plant death. Halotolerant plant growth-promoting rhizobacteria (PGPR) could be a viable alternative for tomato plants growing in arid and semi-arid environments. The aim of this research was to isolate halotolerant plant growth promoting *Bacillus* sp. to promote tomato (*Lycopersicon esculentum* Mill.) growth and salt stress resistance. 107 PGPR strains were isolated from the rhizospheres of ‘Kesudo’ (*Butea monosperma* Lam.), ‘Kawaria’ (*Cassia tora* L.), and ‘Arjun’ (*Terminalia arjuna* Roxb.) plants to test their plant growth promoting abilities, including indole-3-acetic acid, phosphate solubilization, siderophore production, and ACC deaminase activity. Five bacterial strains (*Bacillus pumilus* (NCT4), *Bacillus firmus* (NCT1), *Bacillus licheniformis* (LCT4), *Bacillus cereus* (LAT3), and *Bacillus safensis* (LBM4)) were chosen for 16S rRNA on the basis of PGPR traits. Compared to PGPR untreated plants, tomato plants developed from PGPR-treated seeds had considerably increased germination percentage, seedling growth, plant height, dry weight, and leaf area. As comparison to PGPR non-inoculated plants, salt-stressed tomato plants treated with PGPR strains had higher levels of total soluble sugar, proline, and chlorophyll as well as higher levels of SOD, CAT, APX, and GR activity. PGPR-inoculated salt-stressed tomato plants had lower MDA, sodium, and chloride levels than non-inoculated plants. In addition, magnesium, calcium, potassium, phosphorus, and iron levels were higher in PGPR treated plants when subjected to salt stress. These results indicate that halotolerant PGPR strains can increase tomato productivity and tolerance to salt stress by removing salt stress’s negative effects on plant growth.

## 1 Introduction

Any government has significant difficulties as a result of the rising population and food demand, which transfers the focus to agricultural practices and improving yield improvement. Different agro-ecosystems’ varying climatic conditions, edaphic elements, farming practices, and management strategies are the main determinants of increased crop productivity, which is highly intricate (Shah and Wu, 2019). Crop productivity is hindered by a number of abiotic conditions, including temperature, salinity stress, drought, soil pH, heavy metals, and the use of pesticides and chemical fertilizers (Wei et al., 2020). Salinity stress is considered a serious danger to agricultural production among all of these (Isayenkov and Maathuis, 2019). In arid and semi-arid areas, soil salinity is a major environmental issue that causes imbalanced osmotic stress and reduced plant growth (Cicek and Çakirlar, 2002). Salinity can affect all vegetable crops at varying degrees (Shannon and Grieve, 1998). Salinity can have varied effects on all vegetable crops, reducing crop growth and output *via* altering morphological and physiological processes (Shahbaz et al., 2012). Vegetable growth is impacted by salt stress because of the osmotic or water-deficit effect, toxic salt buildup in shoots, nutritional imbalances, or a combination of these factors (Läuchli and Grattan, 2017).

Many aspects of plant metabolism are affected by salinity stress, and as a consequence, yield and growth are diminished. A high salt content in a soil solution may hinder plant growth either through osmotic inhibition of root water uptake or through particular ion effects. Salinity raises the uptake of Na^+^, which reduces the uptake of Ca^2+^ and K^+^ (Yildirim et al., 2006). Surplus Na^+^ can cause metabolic changes in processes that require low Na^+^ and high K^+^ or Ca^+2^ for optimal function (Marschner, 1995). Cl^-^ uptake and buildup may impair photosynthetic function by reducing nitrate reductase activity (Xu et al., 2000). When a cell’s ability to accumulate salts is depleted, salts accumulate in the intercellular space, causing cell dehydration and demise (Sheldon et al., 2017). Higher saline levels cause the growth rate of the leaf area to slow down, as does the rate of leaf production and leaf size, ultimately resulting in the death of the plant (Suárez and Medina, 2005).

Among the many methods and techniques employed to lessen the detrimental effects of salinity stress on crop production are plant genetic engineering, the use of salt-resistant varieties, organic matter conditioners, and salt stress mitigation substances (Zhang et al., 2000). Salinity, however, is a challenging issue that scientists are working to resolve by creating more practical and affordable solutions that are straightforward to use. These helpful bacteria cause biochemical and morphological changes in the plants once they are inoculated with them, increasing the plants’ resistance to abiotic stress (Kerbab et al., 2021). Due to the rise of more extreme climatic circumstances, inoculating plants with PGPB (plant growth promoting bacteria) can be an efficient way to enhance plant growth by increasing plant resilience to abiotic stresses (Redondo-Gómez et al., 2021). Plant growth promoting rhizobacteria (PGPR) can work directly by improving nutrient intake from the environment or indirectly by lowering plant diseases. Moreover, PGPR can defend plants from the harmful effects of environmental stresses such as drought, salinity, flooding, heavy metals, and phytopathogens (Mayak et al., 2004; Yildirim et al., 2006). The impact of PGPR on the growth of lettuce, bean, pepper, and canola under salinity stress was checked, and the detrimental effects of salinity were mitigated (Glick et al., 1997).

Inoculation of plants with 1-aminocyclopropane-1-carboxylic acid (ACC) deaminase containing PGPR produced increased chlorophyll in maize and lettuce (Glick et al., 1997; Han and Lee, 2005). Numerous PGPRs have the ability to solubilize phosphorus and produce siderophores, enabling host plants to effectively absorb P and Fe-derived nutrients from the soil (Dodd and Perez-Alfocea, 2012). One of the most prevalent taxa of isolated endophytes among PGPRs is *Bacillus*, which offers a sustainable and environmentally acceptable method of plant growth promotion (PGP) through a several processes, including hormone synthesis, nutrient solubilization, and plant bio-protection (Shafi et al., 2017). Plant growth promoting bacteria that produce IAA, such as *Bacillus* species OSU-142, *Paenibacillus polymyxa* RC05, *Pseudomonas putida* RC06, and *Rhodobacter capsulatus* RC04, benefit plant growth development and nutrition uptake (Cakmakci et al., 2007). MDA was harmfully affected by salinity stress and significantly increased by 234.6% as compared to the non-saline control in *Acacia gerrardii* Benth. But inoculation with *Bacillus subtilis* (BERA 71) reduced the harmful effects of salinity on MDA (Hashem et al., 2016). Additionally, it has been observed that rhizobacteria regulate plant antioxidant enzymes including SOD, CAT, and APX to promote salt tolerance in plants (Yilmaz and Kulaz, 2019).

After potatoes, the tomato (*Lycopersicon esculentum* Mill.) is the second-most significant vegetable crop in the world. Due to their well-balanced composition of vitamins (A, B1, B2, B6, C, E, K, biotin, folic acid, nicotinic acid, and pantothenic acid), minerals (potassium, calcium, phosphorus, iron, and zinc), and antioxidants (carotenoids and polyphenolic compounds), tomatoes have an outstanding nutritional profile (Sharma et al., 2009). By altering the activity of crucial enzymes and the amounts of gibberellin (GA), salinity stress slows down and lowers the rate of germination of tomato seeds, respectively (Singh et al., 2012; Tanveer et al., 2020). Salinity stress has three-fold impacts, this results in ion imbalance, toxicity, and a reduction in water potential (La Pena and Hughes, 2007). A PGPR strain of *Achromobacter piechaudii* ARV8 has been shown to reduce salt stress in tomato plants (Mayak et al., 2004).

According to the Food and Agriculture Organization of the United Nations, soil salinity had an impact on more than 833 million hectares of land in 2021. It is estimated that more than 10% of agricultural land is affected by salinity, which poses a significant threat to global food security. Many countries in the Pacific, North Africa, South America, and the Middle East are the worst-affected regions (FAO, 2021). Approximately 2.1% of India’s land surface, or 6.727 million hectares, is impacted by salt, of which 2.956 million hectares are salty and the remaining 3.771 million hectares are sodic (Arora and Sharma, 2017). About 75% of the soils in the country are affected by salt, and the largest states with salt-affected soils are Gujarat (2.23 million hectares), Uttar Pradesh (1.37 million hectares), Maharashtra (0.61 million hectares), West Bengal (0.44 million hectares), and Rajasthan (0.50 million hectares) (Mandal et al., 2018). Nowadays, because of faulty and extensive irrigation practices, much of Gujarat’s agricultural land and coastal land have become saline. The hasty application of various pesticides and agrochemicals has exacerbated the situation.

The present study was carried out to discover the behavior of the selected PGPR strains under salinity stress conditions as well as their role in tomato growth enhancement. Thus, the present study was undertaken. (a) Isolate bacteria with plant growth-promoting traits, (b) Identify and characterize the isolated bacteria based on biochemical characteristics, as well as confirm the bacterial genera through 16S rRNA sequence analysis, and (c) Understand the physiological and biochemical changes that occur after PGPR inoculation in the plant’s rhizosphere during salt stress alleviation.

## 2 Materials and methods

### 2.1 Isolation and identification of microorganisms

#### 2.1.1 Soil sample collection

Three plants were chosen for the isolation of bacteria from the rhizosphere of ‘Kesudo’ (*Butea monosperma* Lam), ‘Kawaria’ (*Cassia tora* L.), and ‘Arjun’ (*Terminalia arjuna* Roxb.) from the Little Rann of the Kutch in Gujarat (23°58′30″N, 70°12′19″E). By uprooting the root system, rhizosphere soil samples were carefully taken at a depth of 10 cm. They were then placed in a cold box for transportation to the laboratory, where they were kept at 4°C.

#### 2.1.2 Bacterial isolation

In 50 ml Erlenmeyer flasks, 2 gm of soil were suspended in 20 ml of sterile distilled water to create soil suspensions. Erlenmeyer flasks were incubated for an hour at 150 rpm in an orbital shaker. 1 ml of the soil suspension and 9 ml of sterile distilled water were combined in glass tubes. By adding 1 ml of the suspension to 9 ml of sterile distilled water at 10^-1^ to 10^-6^ dilutions, serial dilutions were made. Inoculation of plates was done using these dilutions. 200 μl aliquots from various dilutions were transferred and spread on 5% NaCL supplemented nutrient agar plates, luria agar plates, minimal agar plates, and tryptone soya agar plates. All of these plates were incubated in an incubator at 37°C for 24 hours, and morphologically unique colonies that developed on the medium were separated and subcultured for future investigations. A total of 107 isolates were obtained, and slants were used to prepare and maintain the pure cultures of these isolates in their respective media. These 107 bacterial isolates were kept in the refrigerator at 4°C for future use.

#### 2.1.3 Gram’s staining

On a clean glass slide, smears of each bacterial isolate were made separately, dried, and then heat-fixed. The smear was treated with one drop of crystal violet solution and left to react for 45 seconds. The additional stain was removed using sterile water. One drop of Gram’s iodine solution was then applied for 45 seconds. It was then rinsed with water before being submerged for one minute in 100 ml of absolute alcohol. After that, a drop of the counter stain safranin was put to the smear and left to react for one minute. It was gently cleaned with sterilized water, dried by air, placed in glycerin, and examined under oil immersion.

#### 2.1.4 Plant growth promoting traits

##### 2.1.4.1 IAA production

Bacterial isolates were grown for 48 hours at 30°C in nutrient broth containing 500 g/ml DL-tryptophan. Centrifuging was done on fully developed cultures at 4°C for 10 minutes at 10,000 rpm. IAA estimation was done using the supernatant. After that, the orthophosphoric acid (2 drops) and Salkowski reagent (4 ml) were mixed with the supernatant (2 ml). Pink colour manifestation implies IAA production. At 560 nm, the optical density was determined (Bric et al., 1991).

##### 2.1.4.2 Determination of phosphate solubilization

Isolates of bacteria were grown in 50 ml of Pikovskaya’s broth containing 100 mg of tricalcium phosphate, and the amount of soluble phosphorus released on the seventh day after inoculation was calculated. In order to calculate the amount of soluble phosphorous, the culture media was centrifuged for 10 minutes at 10,000 rpm (Olsen, 1954).

##### 2.1.4.3 Estimation of siderophore production

To produce siderophore in 1 litre of distilled water, a small modification was made to the succinate (iron-free) medium. 0.5 ml of the old culture of each test isolate was added to 100 ml of medium in flasks and incubated at 30°C for 72 hours on a rotary shaker. Cell-free supernatant was obtained after centrifugation at 10,000 rpm for 20 minutes at 4°C. The supernatant was used for the estimation of siderophore.

To measure siderophores, the CAS (Chrome azurol S) liquid assay method was employed (Schwyn and Neilands, 1987). The CAS assay solution was combined with 0.5 ml of 72-hour-old cell-free supernatant, and 10 μl of shuttle solution was then added. After 10 minutes at room temperature, the colour intensity of the solution was measured with a UV-VIS spectrophotometer at 630 nm against a reference. A decrease in blue colour as expressed in percent siderophore units (% SU) was seen as a result of siderophore synthesis.

##### 2.1.4.4 ACC deaminase activity

A modified version of Honma and Shimomura (1978) method, which measures the amount of α-ketobutyrate released during ACC hydrolysis, was used to quantify the activity of ACC deaminase. The amount of μmol of α-ketobutyrate generated by this reaction was determined by comparing the sample’s absorbance at 540 nm to a standard curve of α-ketobutyrate with concentrations between 0.1 and 1 μmol.

#### 2.1.5 Molecular identification and phylogenetic tree generation of bacterial isolates

The most efficient bacterial isolates were identified molecularly by sequencing their 16S rRNA gene. Out of 107 bacterial strains, five were chosen for 16S rRNA analysis based on PGPR traits. Bacterial isolates’ 16S rRNA sequences have been deposited into the GenBank database. The BLAST search tool was used to look for nucleotide sequence homology in the bacteria’s 16S region. To align highly homologous sequences and generate a neighbour-joining tree, ClustalW and MEGA version 11.0 were used.

### 2.2 The effect of PGPR inoculation on tomato physiological and biochemical parameters under saline conditions

#### 2.2.1 Preparation of the PGPR strain and inoculum

In this study, five different PGPR strains were used: *Bacillus pumilus* NCT4, *Bacillus firmus* NCT1, *Bacillus licheniformis* LCT4, *Bacillus cereus* LAT3, and *Bacillus safensis* LBM4. The PGPR strain’s active cultures were prepared using nutrient broth and luria broth.

#### 2.2.2 Plant material and study area

Seeds of tomato S-22 (*Lycopersicon esculentum* Mill.) (physical purity: minimum 98%, inert matter: maximum 2%, moisture: maximum 6%, pure seed: minimum 98%, and germination: minimum 70%) were collected on January 23, 2022, from the R.K. seed farms (Regd.), Delhi. The entire experiment was carried out in a greenhouse at Hemchandracharya North Gujarat University’s botanical garden in Patan (23°51’ N Latitude, 72°07’ E Longitude) in Gujarat. For seedling emergence and growth, a nearby agriculture field’s top 15 cm of surface soil (vertisol), which predominates in Gujarat’s northern region, was obtained.

#### 2.2.3 Salinization of soil

The collected surface soil was autoclaved, allowed to air-dry, and then passed through a 2 mm mesh. The 3 kg of soil was then thoroughly mixed with 7.8 g of sodium chloride (NaCl), resulting in an interstitial soil water salinity of 4 dsm^-1^. To measure soil salinity, a soil suspension in distilled water with a soil:water ratio of 1:2 was prepared (Patel et al., 2009). After a thorough shaking, the soil suspension was left to stand for the night. Then, a conductivity metre (Systronic; Model 307) was used to measure the soil suspension’s conductivity.

#### 2.2.4 Experimental design

A total of 3 kg of soil was filled in 10 polythene bags (20.5 cm wide and 41 cm long) for each of the twelve treatments. After that, 120 bags were placed in an uncontrolled greenhouse with natural light and temperature. Healthy tomato seeds were surface sterilized for 1 minute with 0.1% Mercuric chloride (HgCl_2)_ and rinsed six times with sterile distilled water before Biopriming in the appropriate active bacterial culture for 30 minutes. Seeds for control plants were soaked in sterile water for the same amount of time. The seeds were then dried in the shade for 30 minutes. On January 24, 2022, twenty seeds were gently pressed to a depth of approximately 10-15 mm in each bag after drying in the shade. Then, except for the control treatment, 30 ml of active culture was poured to each bag. On alternate days, 100 ml of tap water was given to moisten the soil’s surface. The experiment was repeated three times in a completely randomized block design with ten replicates.

#### 2.2.5 Seedling growth

Two of the seedlings that established first were kept in each bag, while others were uprooted as they appeared. After two months, the experiment was stopped. For each treatment, 20 plants were grown, and afterward they were cleaned with tap water to get rid of any soil that had stuck to the roots. Each seedling’s morphological characteristics were noted. Up to 30 days following seeding, seed germination (%) was recorded. Using a scale, the height of the plant shoots was measured from the plant’s tip to the stem’s end using a scale, the length of the plant’s roots was calculated from the collar region to the end of the root. A weighing machine was used to measure the fresh weight of the shoots and roots right after harvest. Before weighing the fresh weight of the shoot and root, extra moisture on them was blotted using tissue paper. After 5 days of drying in a hot air oven at 40°C, when a constant weight was attained, the dry weight of the shoot and root was measured using a weighing machine. On graph paper, the leaf area was marked out.

#### 2.2.6 Biochemical parameters

##### 2.2.6.1 Organic solutes (soluble sugars and proline)

The total soluble sugar concentration was estimated using the phenol-sulfuric acid method **(** Krishnaveni et al., 1984). 100 mg of leaves were hydrolyzed with 5 ml of 2.5 N HCl in a boiling water bath for 3 hours, and the reaction was then neutralized with solid sodium carbonate until the effervescence subsided. Then, the volume was raised to 100 ml, and it was centrifuged. Following that, 0.1 and 0.2 ml supernatant aliquots were obtained and increased to 1 ml in separate test tubes. The next step was to add 1 ml of phenol solution and 5 ml of 96% sulfuric acid to each test tube, shake them vigorously, and then place them in a water bath at 25 to 30°C for 20 minutes. At 490 nm, the chromophore was read. The total amount of carbohydrates was determined using the glucose standard curve.

The amount of proline in a sample was measured according to Patel et al. (2014). 500 mg of plant leaves were ground in 10 ml of 3% sulfosalicylic acid, and the mixture was centrifuged at 10,000 g for 10 minutes to extract the proline. In a test tube, an aliquot of 2 ml of supernatant was placed, and an equivalent volume of freshly made ninhydrin solution was added. The tubes were incubated for 30 minutes at 90°C in a water bath. The reaction was stopped using an ice bath after incubation. Then, while stirring constantly for 15 minutes, the reaction mixture was extracted with 5 ml of toluene. The tubes were left in the dark for 20 minutes in order to separate the supernatant of the toluene and aqueous phases. The toluene phase was carefully collected into a test tube, and the absorbance was then recorded at 520 nm. The concentration of proline was determined from a standard curve using the equation (μg proline in extract/111.5)/g of sample = μmol g^-1^ of fresh tissue.

##### 2.2.6.2 Total chlorophyll content

The content of chlorophyll was determined according to Arnon (1949). Approximately 1 g of leaves were chopped into small pieces and homogenized with 80 percent (V/V) acetone in a precooled mortar and pestle. A small amount of CaCO_3_ was added during the grinding. After centrifuging the extract for 15 minutes at 3000 rpm, it was diluted with 80 percent (V/V) acetone to make up to 25 ml. In a spectrophotometer, the OD of the clear solution was taken at 645 nm and 663 nm against a blank of 80% acetone. The following equation was used to determine the levels of chlorophyll ‘a’ and chlorophyll ‘b’:

Chlorophyll ‘a’ (μ/g/ml) = (12.7 × O.D. at 663 nm) – (2.69 × O.D. at 645 nm)

Chlorophyll ‘b’ (μ/g/ml) = (22.9 × O.D. at 645 nm) – (4.08 × O.D. at 663 nm)

Total chlorophyll (μ/g/ml) = (20.2 ×O.D. at 645 nm) + (8.02 × O.D. at 663 nm)

The content of chlorophyll was expressed as mg chlorophyll per gram fresh weight of the tissue.

##### 2.2.6.3 Lipid peroxidation

Lipid peroxidation was estimated as the amount of malondialdehyde (MDA) determined by the TBA reaction as described by Heath and Packer (1968) with some modifications. 2 ml of 1% TCA were used to homogenize 200 mg of plant leaves before they were centrifuged at 10,000 × g for 15 minutes. 1 ml of the supernatant aliquot, 2 ml of 20% w/v TCA, and 2 ml of 0.5% TBA were mixed, and incubated at 95°C for 30 minutes, followed by a quick transfer to an ice bath in order to stop the reaction. The absorbance at 532 nm was measured following centrifugation at 10,000 × g for 5 min. The value at 532 nm was deducted from the value of non-specific absorbance at 600 nm. MDA concentration was determined from the extinction coefficient at 155 mM^-1^cm^-1^and it was defined as µmol g^-1^ fresh weight MDA.

##### 2.2.6.4 Antioxidant enzymes

To prepare plant leaf extractions for analysis, 200 mg of plant material was homogenized in 2 ml of 0.2 M potassium phosphate buffer (pH 7.8 with 0.1 mM EDTA). At 4°C, the homogenate was centrifuged for 20 minutes at 15,000 × g. After that, the tissue extract was kept at -20°Cand used within 48 hours to estimate different antioxidant enzymatic activities.

A modified NBT (nitro blue tetrazolium) method developed by Beyer and Fridovich (1987) was used to measure the activity of superoxide dismutase (SOD). A 2 ml portion of the assay reaction mixture, which includes 50 mM phosphate buffer (pH 7.8), 2 mM EDTA, 9.9 mM L-methionine, 55 mM NBT, and 0.025% Triton X-100, was put into a test tube. After that, 40 μl of the diluted (×2) sample and 20 μl of 1 mM riboflavin were added, and then the reaction was started by illuminating the sample under a 15-W fluorescent tube. For the 10minute exposure, the test tubes were placed in a box that was lined with aluminum foil and placed about 12 cm away from the light source. The same reaction mixture was also contained in duplicate tubes, which were kept in the dark and utilized as blanks. The amount of enzyme per milligram of protein sample that causes 50% inhibition of the rate of NBT reduction at 560 nm was defined as one unit of SOD.

The activity of catalase (CAT) was measured according to Aebi (1984). A 3 ml assay mixture contained 10 mM H_2_O_2_ and 2 ml of plant leaf extract that had been 200 times diluted in a 50 mM potassium phosphate buffer with a pH of 7.0. The reduction in absorbance at 240 nm signaled the breakdown of hydrogen peroxide. The enzyme activity was calculated using the extinction coefficient of H_2_O_2_ (40 mM^-1^ cm^-1^ at 240 nm).

The activity of ascorbate peroxidase (APX) was determined using the method of Nakano and Asada (1981). 0.5 mM H_2_O_2_, 50 mM potassium phosphate buffer (pH 7.0), and 10 μl of plant leaf extract were all present in one ml of the assay mixture. To start the reaction, H_2_O_2_ was added last, and the drop in absorbance was measured for 3 minutes. The reduction in absorbance at 290 nm brought on by the oxidation of ascorbate during the reaction was used to determine the ascorbate peroxidase activity. The enzyme activity of APX was calculated using a reduced ascorbate extinction coefficient of 2.8 mM^-1^ cm^-1^.

According to Smith et al. (1988) glutathione reductase (GR) activity was determined. A 10 μl aliquot of leaves extract was used in the assay along with 0.1 mM NADPH, 0.75 mM DTNB (Ellman’s reagent; 5,5’-dithiobis-(2-nitrobenzoic acid)), and 1 mM GSSG in a total of 1 ml of assay volume. To begin the reaction, GSSG was added last, and the absorbance increase was measured for 3 minutes. The increase in absorbance at 412 nm was detected as GSH reduced DTNB to TNB during the process measured. The activity of glutathione reductase was calculated using the extinction coefficient of TNB (14.15 mM ^-1^ cm^-1^).

### 2.3 Mineral analysis of plant materials

On the leaves, mineral analyses were performed. A mortar and pestle were used to grind the plant leaves. Plant samples were evaluated in three subsamples. After triacid digestion (HNO_3_: H_2_SO_4_: HclO_4_ in the ratio of 10: 1: 4), the concentrations of Na, K, Ca, Fe, and Mg were measured using atomic absorption spectroscopy. The Mohr method was used to determine chloride using K_2_Cr_2_O_7_ as an indicator in a titration of Cl ions with AgNO_3_ standard solution (Johnson and Ulrich, 1959; Kacar and Inal, 2008). The chlorostannous molybdophosphoric blue colour technique in sulfuric acid was used to estimate the phosphorus level (Piper, 1944).

### 2.4 Statistical analysis

Multivariate cluster analysis was performed to construct a dendrogram based on the similarity matrix of physico-chemical parameters using the paired group (UPGMA) method with arithmetic averages and Bray-Curtis similarity index. Non-metric multidimensional scaling was done to group the treated and non-treated plants on the basis of similarity in different physico-chemical parameters. To determine the significant relationships between the measured parameters, principal component analysis was applied to all of the parameters. To evaluate the relationships between various physico-chemical parameters, Pearson’s correlation coefficient analyses were performed. To find significant variation between means, comparison and similarity groups of all measured parameters were performed using two-way ANOVA. These analyses were performed carried out using PAST: Palaeontological Statistics software package version 4.05 (Hammer et al., 2001).

## 3 Results

### 3.1 Isolation and identification of bacterial strain

There were 107 different bacterial strains detected in the soil from the rhizospheres of the “Kesudo” (*Butea monosperma* Lam.), “Kawaria” (*Cassia tora* L.), and “Arjun” (*Terminalia arjuna* Roxb.). These bacteria were both gram positive and gram negative.

#### 3.1.1 Plant growth promoting traits

The traits that promote plant growth were evaluated in all 107 strains and found in 5 highly potent PGPR strains (NCT4, NCT1, LCT4, LAT3, and LBM4). Indole-3-acetic acid (IAA) and siderophore are produced by all five strains. Except for the LBM4 strain, all four strains were able to solubilize phosphate, and except for LAT3 and LBM4 strains, all three strains produced ACC deaminase (**Table 1**).

**Table 1:** Plant growth-promoting rhizobacteria (PGPR) traits of the selected isolates.

Isolates	IAA production (μg/ml)	Phosphate solubilization (mg of P released from 100 mg of tricalcium phosphate)	% Siderophore Units (SU)	ACC deaminase activity (nmoL α- ketobutyrate mg^-1^ h^-1^)	Gen Bank Accession No.
*Bacillus pumilus* NCT4	42.5	11.69	48.7	354.8	KF853108
*Bacillus firmus* NCT1	45.7	6.17	41.2	316.5	KF853131
*Bacillus licheniformis* LCT4	27.3	5.85	56.1	641.2	KF853123
*Bacillus cereus* LAT3	15.6	2.08	49.3	ND	KF853105
*Bacillus safensis* LBM4	5.3	ND	38.2	ND	KJ883295

ND, Not Detected.

#### 3.1.2 Molecular identification and phylogenetic tree generation

**Figure 1:**
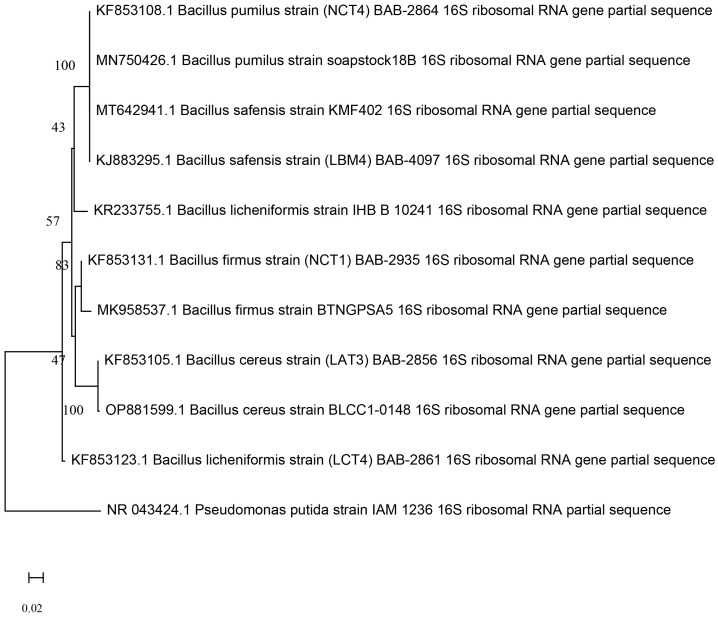
Phylogenetic relationship among the KF853108 *Bacillus pumilus* strain (NCT4), KJ883295 *Bacillus safensis* strain (LBM4), KF853123 *Bacillus licheniformis* strain (LCT4), KF853105 *Bacillus cereus* strain (LAT3), KF853131 *Bacillus firmus* strain (NCT1) and other type strains based on 16S rRNA gene sequences generated by neighbor end joining method.

Five of the best performing strains according to their PGPR traits were from the *Bacillus* group. The 16S rRNA sequence were submitted to NCBI for identification of strain using the BLAST database and it was found that, isolate coded as NCT4 showed 100% sequence similarities with *Bacillus pumilus*18B (MN750426), NCT1 showed 98.64% sequence similarities with *Bacillus firmus* BTNGPSA5 (MK958537), LCT4 showed 98.8% sequence similarities with *Bacillus licheniformis* IHB B 10241 (KR233755), LAT3 showed 100% similarities with *Bacillus cereus* BLCC1-0148 (OP881599), and LBM4 showed 99.78% similarities with *Bacillus safensis* KMF402 (MT642941). All sequences were submitted to GeneBank which are accessible through accession number KF853108, KF853131, KF853123, KF853105, and KJ883295 respectively. Evolutionary relationships between the isolates generated by the Mega (version 11) programme (**Figure 1**). Next to the branches is the percentage of replicate trees in which the related taxa clustered together in the bootstrap test (1000 repetitions).

### 3.2 Effect of PGPR on physiological parameters of plant under saline conditions

In this work, the impact of PGPR on tomato plant growth in normal and saline conditions was investigated. Each PGPR strain significantly enhanced the tomato plants’ root length, shoot height, leaf area, and germination percentage. Under 4 dsm^-1^ saline stress conditions, treated plants with NCT4, NCT1, LCT4, LAT3, and LBM4 had considerably larger shoot heights than untreated plants. As well, under 4 dsm^-1^ saline stress conditions, treated plants with NCT4, NCT1, and LCT4 had considerably longer plant roots than untreated plants (**Table 2**).

**Table 2:** Effects of plant growth-promoting rhizobacteria on the shoot height, root length, leaf area and germination % of tomato plants under saline conditions.

NaCl concentration	Treatment	Shoot height (cm)	Root length(cm)	Leaf Area (cm^2^)	Germination (%)
0 dsm^-1^	Non-inoculated	45.76 ± 2.4a	12.44 ± 1.7a	6.91 ± 0.5a	79 ± 6.4a
	NCT4	69.28 ± 4.8c	19.52 ± 1.8b	8.71 ± 0.9b	95 ± 5.4b
	NCT1	66.4 ± 3.4bc	17.76 ± 1.7b	8.29 ± 0.6b	93 ± 6.7b
	LCT4	60.32 ± 5.1bc	16.16 ± 2.1b	7.71 ± 0.7ab	86 ± 4.5ab
	LAT3	54.24 ± 5.3b	14.56 ± 1.0ab	7.32 ± 0.5ab	84 ± 3.8ab
	LBM4	51.71 ± 3.1b	14.24 ± 0.9ab	7.13 ± 0.6ab	83 ± 3.1ab
4 dsm^-1^	Non-inoculated	37.9 ± 1.1a	10.68 ± 1.2a	5.72 ± 0.3a	63± 3.4a
	NCT4	57.35 ± 3.0c	16.15 ± 1.4c	7.18 ± 0.4b	76 ± 4.6b
	NCT1	54.45 ± 3.2bc	14.67 ± 1.2bc	6.85 ± 0.9ab	74 ± 4.3b
	LCT4	49.94 ± 4.2b	13.37 ± 1.3b	6.37 ± 0.4a	68 ± 3.3ab
	LAT3	45.49 ± 4.5b	12.5 ± 1.4ab	5.90 ± 0.2a	67 ± 3.2ab
	LBM4	42.75 ± 2.1b	11.77 ± 0.6ab	5.83 ± 0.4a	66 ± 2.6a

Data were analysed using the One-way ANOVA Tukey’s multiple range test (P<0.05). Different small letters have significant differences.

Moreover, in non-saline environments, the shoot height of the plant was significantly greater in NCT4, NCT1, LCT4, LAT3, and LBM4 treated plants than in non-inoculated plants, and the root length was significantly greater in NCT4, NCT1, and LCT4 treated plants than in non-inoculated plant. However, when treated with various microorganisms, tomato plant leaf area was also affected in both growing conditions (saline 4 dsm^-1^ and non-saline 0 dsm^-1^). Tomato leaf area increased significantly when treated with the NCT4 strain under saline conditions, just as it increased considerably when treated with the NCT4 and NCT1 strains under non-saline conditions. In both saline and non-saline conditions, the NCT4 and NCT1 strains greatly boosted the tomato plants’ germination percentage (**Table 2**).

Each of the PGPR strains significantly increased the tomato plant’s shoot fresh weight, shoot dry weight, root fresh weight, and root dry weight. Under 4 dsm^-1^ salt stress conditions, treated plants with NCT4, NCT1, LCT4, LAT3, and LBM4 had considerably higher shoot fresh weights than untreated plants. As well, the shoot dry weight of the plant was significantly greater in NCT4, NCT1, LCT4, LAT3, and LBM4 treated plants than in untreated plants under the4 dsm^-1^ saline stress condition. Additionally, in non-saline conditions, the NCT4-treated plant’s shoot fresh weight was considerably higher than the untreated plant. The root fresh weight of the plant was significantly greater in NCT4 and NCT1-treated plants than in untreated plants under 4 dsm^-1^ saline stress conditions. Additionally, under 4 dsm^-1^ saline stress conditions, the root dry weight of the plant was considerably higher in NCT4 and NCT1 treated plants than in untreated plants. The root fresh weight of the plant was significantly higher in the NCT4 treated plant than the untreated plant in the non-saline condition, and the root dry weight of the plant was significantly higher in the NCT4, NCT1, and LCT4 treated plant than the untreated plant (**Table 3**).

**Table 3:** Effects of plant growth-promoting rhizobacteria on the shoot fresh weight, shoot dry weight, root fresh weight, and root dry weight of tomato plants under saline conditions.

NaCl Concentration	Treatment	Shoot fresh weight (gm)	Shoot dry weight (gm)	Root fresh weight (gm)	Root dry weight (gm)
0 dsm^-1^	Non-inoculated	9.58 ± 0.8a	3.44 ± 0.4a	0.59 ± 0.07a	0.21 ± 0.02a
	NCT4	11.13 ± 1.1b	3.99 ± 0.4a	0.91 ± 0.04b	0.33 ± 0.03c
	NCT1	10.81 ± 0.9ab	3.87 ± 0.4a	0.79 ± 0.08ab	0.28 ± 0.02bc
	LCT4	10.54 ± 0.9ab	3.77 ± 0.4a	0.71 ± 0.11a	0.25 ± 0.01b
	LAT3	10.21 ± 1.1a	3.64 ± 0.4a	0.66 ± 0.08a	0.23 ± 0.03ab
	LBM4	10.18 ± 0.8a	3.62 ± 0.2a	0.64 ± 0.10a	0.22 ± 0.01a
4 dsm^-1^	Non-inoculated	5.74 ± 0.5a	2.05 ± 0.1a	0.49 ± 0.03a	0.16 ± 0.01a
	NCT4	9.35 ± 0.8b	3.35 ± 0.2bc	0.74± 0.07b	0.26 ± 0.01c
	NCT1	9.03 ± 0.4b	3.23 ± 0.1bc	0.63± 0.07b	0.22 ± 0.01b
	LCT4	8.85 ± 0.6b	3.17 ± 0.2bc	0.57± 0.08a	0.19 ± 0.02ab
	LAT3	8.56 ± 0.8b	3.06 ± 0.2b	0.53± 0.04a	0.18 ± 0.02a
	LBM4	8.42 ± 0.4b	3.0 ± 0.1b	0.52± 0.05a	0.18 ± 0.03a

Data were analysed using the One-way ANOVA Tukey’s multiple range test (P<0.05). Different small letters have significant differences.

### 3.3 Effect of PGPR in a saline condition on proline and soluble sugar

**Figure 2:**
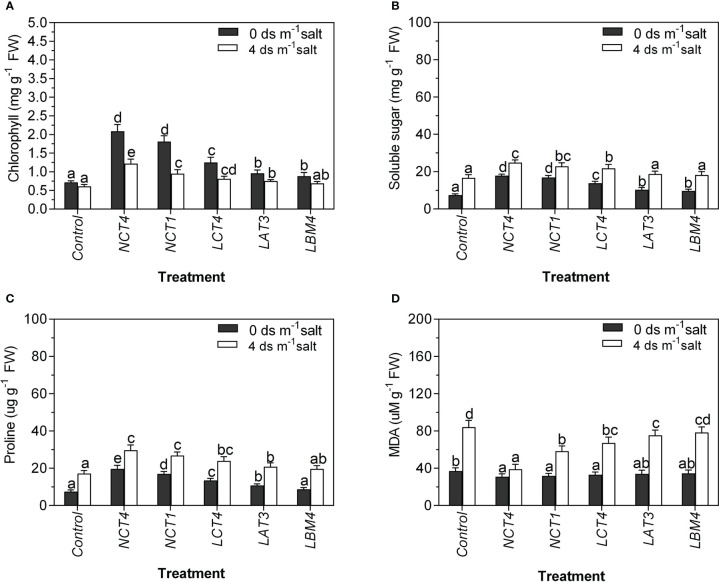
Effects of plant growth promoting rhizobacteria (PGPR) on the **(A)** Chlorophyll **(B)** Soluble sugar **(C)** Proline **(D)** MDA leaf extracts of tomato plant under saline condition. Data were analysed using the One-way ANOVA Tukey’s multiple range test (P<0.05). Different small letters have significant differences.

Common organic solutes in higher plants like proline and soluble sugar build up as a result of stress. Proline and soluble sugar levels in PGPR-treated plants were considerably greater than those in untreated control plants, and inoculated plants accumulated more proline and soluble sugar under saline conditions than non-saline conditions. The soluble sugar content of salinity stressed tomato plants inoculated with three strains, NCT4, NCT1 and LCT4, was significantly increased, whereas in normal conditions, plants inoculated with strains NCT4, NCT1, LCT4, LAT3, and LBM4 were significantly increased. However, the proline content of salt stressed and non-salt stressed tomato plants inoculated with four strains, NCT4, NCT1, LCT4, and LAT3, was significantly increased (**Figure 2**).

### 3.4 Effect of PGPR on chlorophyll content and MDA under saline condition

In both normal and saline conditions, the chlorophyll content and MDA of tomato leaves were measured. Significant differences were found in the chlorophyll content of tomato plants as influenced by PGPR under saline and non-saline conditions. There was a significant raise in the chlorophyll of the leaves of tomato plants in NCT4, NCT1, LCT4, LAT3, and LBM4 treated plants compared to those of untreated plants under non-saline conditions. In contrast, there was a considerable increase in the chlorophyll of tomato plant leaves treated with NCT4, NCT1, LCT4, and LAT3 compared to untreated plants under 4 dsm^-1^ saline (**Figure 2**).

The results of lipid peroxidation of tomato plants as influenced by PGPR in saline and non-saline conditions revealed significant differences. Under non-saline conditions, lipid peroxidation of tomato plant leaves was significantly reduced in NCT4, NCT1, and LCT4 treated plants compared to untreated plants; however, there was no significant effect on lipid peroxidation of leaves in LAT3 and LBM4 treated plants. Under 4 dsm^-1^ saline conditions, lipid peroxidation of tomato plant leaves was significantly reduced in NCT4, NCT1, LCT4, and LAT3 treated plants compared to untreated plants; however, there was no considerable effect on lipid peroxidation of leaves in LBM4 treated plants (**Figure 2**).

### 3.5 Effect of PGPR on the activity of antioxidant enzymes in saline conditions

**Figure 3:**
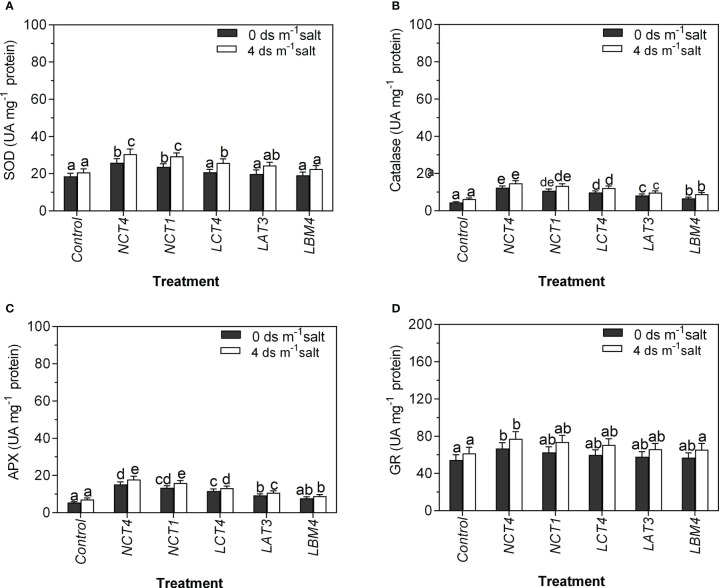
Effects of plant growth promoting rhizobacteria (PGPR) on the antioxidant enzyme activity of leaf extracts of tomato plant under saline condition. **(A)** Superoxide dismutase (SOD) activity **(B)** Catalase activity **(C)** Ascorbate peroxidase (APX) activity **(D)** Glutathione reductase (GR) activity. Data were analysed using the One-way ANOVA Tukey’s multiple range test (P<0.05). Different small letters have significant differences.

In the current work, the activity of four antioxidant enzymes (SOD, CAT, APX, and GR) was determined in leaf extracts from tomato plants grown under normal and saline conditions with and without PGPR inoculation. Inoculation along with all five PGPR strains boosted the activity of all four antioxidative enzymes under these conditions. To be more precise, tomato plants inoculated with NCT4 and NCT1 had considerably higher SOD enzyme activity under the non-saline condition compared to the control condition, and there was no significant effect on LCT4, LAT3, and LBM4 under the non-saline condition. Under 4 dsm^-1^saline condition, there was a considerable increase in SOD of tomato plant leaves in NCT4, NCT1, and LCT4 treated plants compared to untreated plants, but there was no significant effect on SOD of leaves in LAT3 and LBM4 treated plants (**Figure 3**).

Both in saline and non-saline conditions, tomato plants treated with all five strains showed considerably higher catalase enzyme activity. Furthermore, in non-saline conditions, tomato plants inoculated with NCT4, NCT1, LCT4, and LAT3 had much higher APX enzyme activity than control plants, but LBM4 treatment had no discernible impact on leaves’ APX activity. Whereas, under 4 dsm^-1^ saline conditions, compared to untreated plants, all five PGPR-treated tomato plants showed a considerable rise in APX of their leaves (**Figure 3**).

The GR enzyme activity in tomato plants treated with NCT4 was significantly increased in saline and non-saline conditions compared to the control, but there was no significant effect on the GR enzyme activity of leaves in NCT1, LCT4, LAT3, and LBM4 treated plants in non-saline and saline conditions. These results demonstrate that PGPR treated plants’ elevated antioxidant enzyme activity may contribute in their higher salt tolerance (**Figure 3**).

### 3.6 Effect of PGPR on mineral analysis of plant materials under saline condition

**Figure 4:**
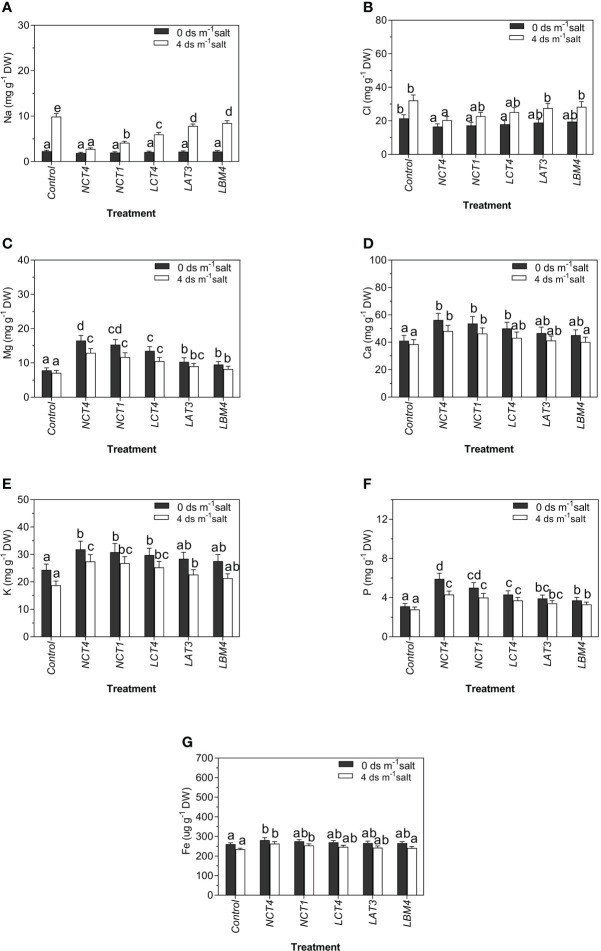
Effects of plant growth promoting rhizobacteria (PGPR) on the **(A)** Na **(B)** Cl **(C)** Mg **(D)** Ca **(E)** K **(F)** P **(G)** Fe of leaf extracts of tomato plant under saline condition. Data were analysed using the One-way ANOVA Tukey’s multiple range test (P<0.05). Different small letters have significant differences.

The effects of PGPR on Na content in tomato plants in saline and non-saline conditions revealed significant differences. There was a considerable decrease in Na content of leaves of tomato plants in NCT4 treated plants compared with untreated plants under non-saline conditions, and there was no significant effect on the Na content of leaves in NCT1, LCT4, LAT3, and LBM4 treated plants under non-saline conditions. Under 4 dsm^-1^ saline conditions, there was a significant decrease in Na content of tomato plant leaves in all five PGPR (NCT4, NCT1, LCT4, LAT3, and LBM4) treated plants compared to untreated plants (**Figure 4**).

Significant differences were noticed in tomato plants with respect to Cl contents influenced by PGPR in saline and non-saline conditions. Under non-saline conditions, the Cl content of tomato leaves was significantly lower in NCT4, NCT1, and LCT4treated plants than in untreated plants, whereas there was no notable effect on the Cl content of leaves in LCT4, LAT3, and LBM4treated plants. Salinity stress conditions of 4 dsm^-1^, the Cl of tomato plant leaves was significantly lower in the NCT4-treated plant than in the untreated plant, but there was no considerable effect on the Cl content of leaves in the NCT1, LCT4, LAT3, and LBM4 treated plants (**Figure 4**).

The results of Mg content of tomato plants as influenced by PGPR under saline and non-saline conditions revealed significant differences. Both in non-saline and saline environments, there was a major increase in Mg content of leaves of tomato plant in all five PGPR (NCT4, NCT1, LCT4, LAT3, and LBM4) treated plants compared to untreated plants (**Figure 4**).

Significant differences were noticed in tomato plants with respect to Ca content as influenced by PGPR under saline and non-saline conditions. Under non-saline conditions, the Ca content of tomato plant leaves was notably higher in NCT4, NCT1, and LCT4 treated plants than in untreated plants, with no significant effect on the Ca content of leaves in LAT3 and LBM4 treated plants. Under 4 dsm^-1^ saline stress conditions, the Ca content of tomato leaves was significantly higher in NCT4 and NCT1 treated plants than in untreated plants, but there was no considerable effect on the Ca content of leaves in LCT4, LAT3, and LBM4 treated plants (**Figure 4**).

The results for K content of tomato plants as influenced by PGPR under saline and non-saline conditions revealed significant differences. Under non-saline circumstances, there was a significant increase in K of tomato leaves in NCT4, NCT1, and LCT4 treated plants compared to untreated plants, but there was no significant effect on K content of leaves in LAT3 and LBM4 treated plants. Under 4 dsm^-1^saline conditions, there was a significant raise in K of tomato leaves in NCT4, NCT1, LCT4, and LAT3 treated plants compared to untreated plants, but there was no significant effect on K of leaves in LBM4 treated plants (**Figure 4**).

Significant differences were noticed in tomato plants with respect to P contents influenced by PGPR both in salinized and non-salinized environments. The P content of leaves of the tomato plant was significantly higher in all five PGPR (NCT4, NCT1, LCT4, LAT3, and LBM4) treated plants than in untreated plants under non-saline and 4 dsm^-1^ saline stress conditions (**Figure 4**).

Significant differences were noticed in tomato plants with respect to Fe contents influenced by PGPR under saline and non-saline conditions. Under conditions that are not salty, the Fe content of tomato plant leaves was notably higher in NCT4 treated plants than in untreated plants, with no significant impact on the Fe content of leaves in NCT1, LCT4, LAT3, and LBM4 treated plants. Under 4 dsm^-1^ saline stress conditions, the Fe content of tomato plant leaves was significantly higher in NCT4 and NCT1-treated plants than in untreated plants, but there was no considerable effect on the Fe content of leaves in LCT4, LAT3, and LBM4-treated plants (**Figure 4**).

### 3.7 Statistical analysis of plant growth and physico-chemical parameters

**Figure 5:**
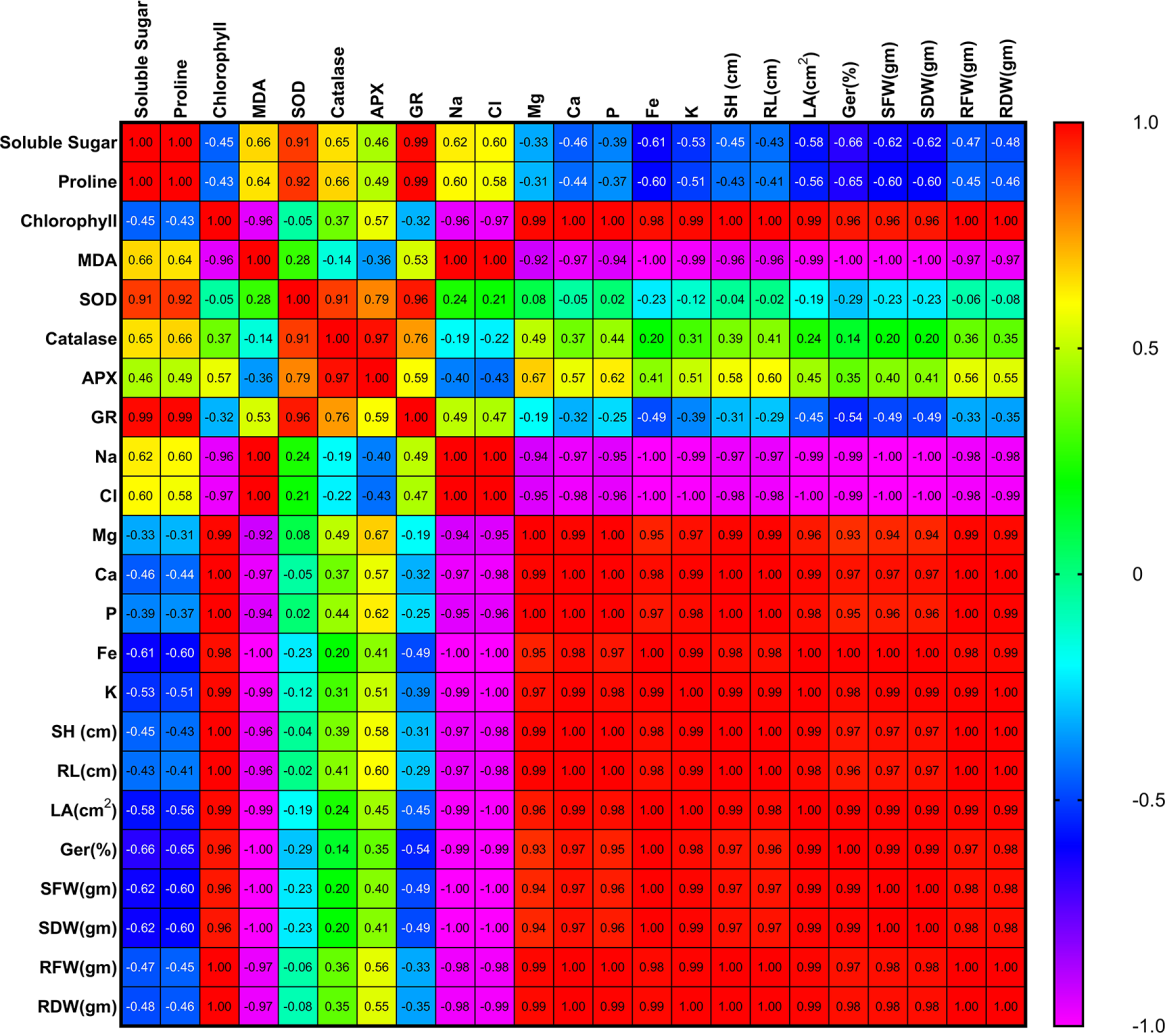
Correlation matrix among the different parameters of tomato plant influenced by salt stress as well as PGPR inoculation. Here, MDA, Malondialdehyde; SOD, Superoxide dismutase; APX, Ascorbate peroxidase; GR, Glutathione reductase; Na, Sodium; Cl, Chloride; Mg, Magnesium; Ca, Calcium; P, Phosphorus; Fe, Iron; K, Potassium; SH, Shoot height; RL, Root length; LA, Leaf area; Ger(%), Germination (%); SFW, Shoot fresh weight; SDW, Shoot dry weight; RFW, Root fresh weight; RDW, Root dry weight.

From the correlation analysis, it was observed that there was positive correlation of soluble sugar and proline content with SOD, catalase, APX and GR which suggests that these parameters help in increasing the activity of these enzymes during stress. There was positive correlation of germination rate with Fe and K content whereas negative correlation of germination rate with MDA and Cl content which suggests that Fe and K promoted germination of plants during stress whereas MDA and Cl inhibited germination of plants during stress. There was negative correlation of Fe and K with MDA, Na and Cl content which suggests that they reduce the effect of these oxidative parameters in treated plants. There was negative correlation of shoot length, root length and leaf area with the chlorine content which inhibited the growth of plants during stress. However, there was positive correlation of shoot length, root length and leaf area with the chlorophyll, Mg, Ca, P, Fe and K content which suggested that there was remarkable growth in treated plants due to the increase in these (chlorophyll, Mg, Ca, P, Fe and K) physico-chemical parameters (**Figure 5** and **Table S1**).

**Figure 6:**
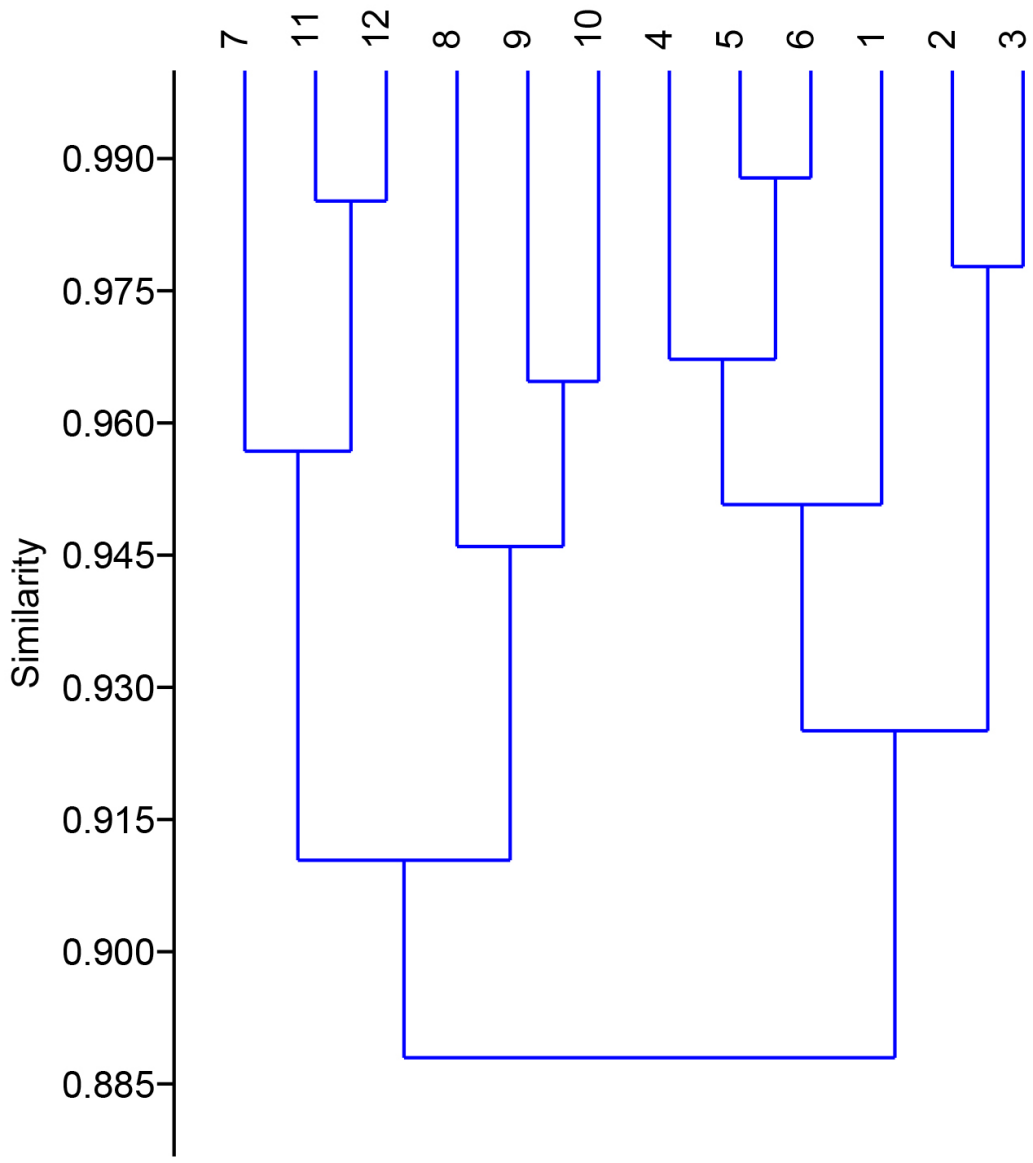
Dendrogram based on Paired group (UPGMA) algorithm using similarity index Bray-curtis for clustering of control and treated plants on the basis of growth and physico-chemical parameters. (1. Control plants 2. NCT4 treated plants 3. NCT1 treated plants 4. LCT4 treated plants 5. LAT3 treated plants 6. LBM4 treated plants 7. Control plants grown in presence of salt 8. NCT4+salt treated plants 9. NCT1+salt treated plants 10. LCT4+salt treated plants 11. LAT3+salt treated plants 12. LBM4+salt treated plants).

The Multivariate cluster analysis was done to detect the similarity between the treated and non-treated plants. Cluster analysis grouped the treated and non-treated plants into four groups on the basis of growth and physico-chemical characteristics. The group A included plants grown in presence of NCT4 and NCT1 culture, that had high germination rate, shoot length, root length, leaf and area. In physico-chemical parameters plants grown in presence of NCT4 and NCT1 had high chlorophyll, Mg, Ca, Fe, P and K content whereas low Na and Cl content. It had moderate MDA, proline and soluble sugar content and moderate enzyme activity of superoxide dismutase (SOD), catalase, Ascorbate peroxidase (APX) and Glutathione reductase (GR) in these plants. Group B included control and plants grown in presence of LCT4, LAT3, and LBM4 culture, that had high germination rate, shoot length, root length, and leaf area which was similar to control plants. In physico-chemical parameters, it had moderate chlorophyll, Mg, Ca, Fe, P, K, MDA, proline and soluble sugar content whereas low Na and Cl content. There was remarkable low enzyme activity of superoxide dismutase, catalase, Ascorbate peroxidase and Glutathione reductase in these plants. Group C included plants grown in presence of NCT4+salt, NCT1+salt and LCT4+salt, that had moderate germination rate, shoot length, root length, and leaf area. In physico-chemical parameters, it had moderate Mg, Ca, Fe, P, K, Na, Cl and chlorophyll content whereas high MDA, proline and soluble sugar content. There was remarkable high enzyme activity of superoxide dismutase, catalase, Ascorbate peroxidase and Glutathione reductase in these plants. Group D included control plants grown in presence of salt and plant grown in presence of LAT3+salt and LBM4+salt, that had low germination rate, shoot length, root length, and leaf area. In physico-chemical parameters, it had low Mg, Ca, Fe, P, K content whereas high Na, and Cl content. It had moderate chlorophyll proline and soluble sugar content whereas high MDA, content. There was moderate enzyme activity of superoxide dismutase, catalase, Ascorbate peroxidase and Glutathione reductase in these plants as compared to other plants (**Figure 6**).

**Figure 7:**
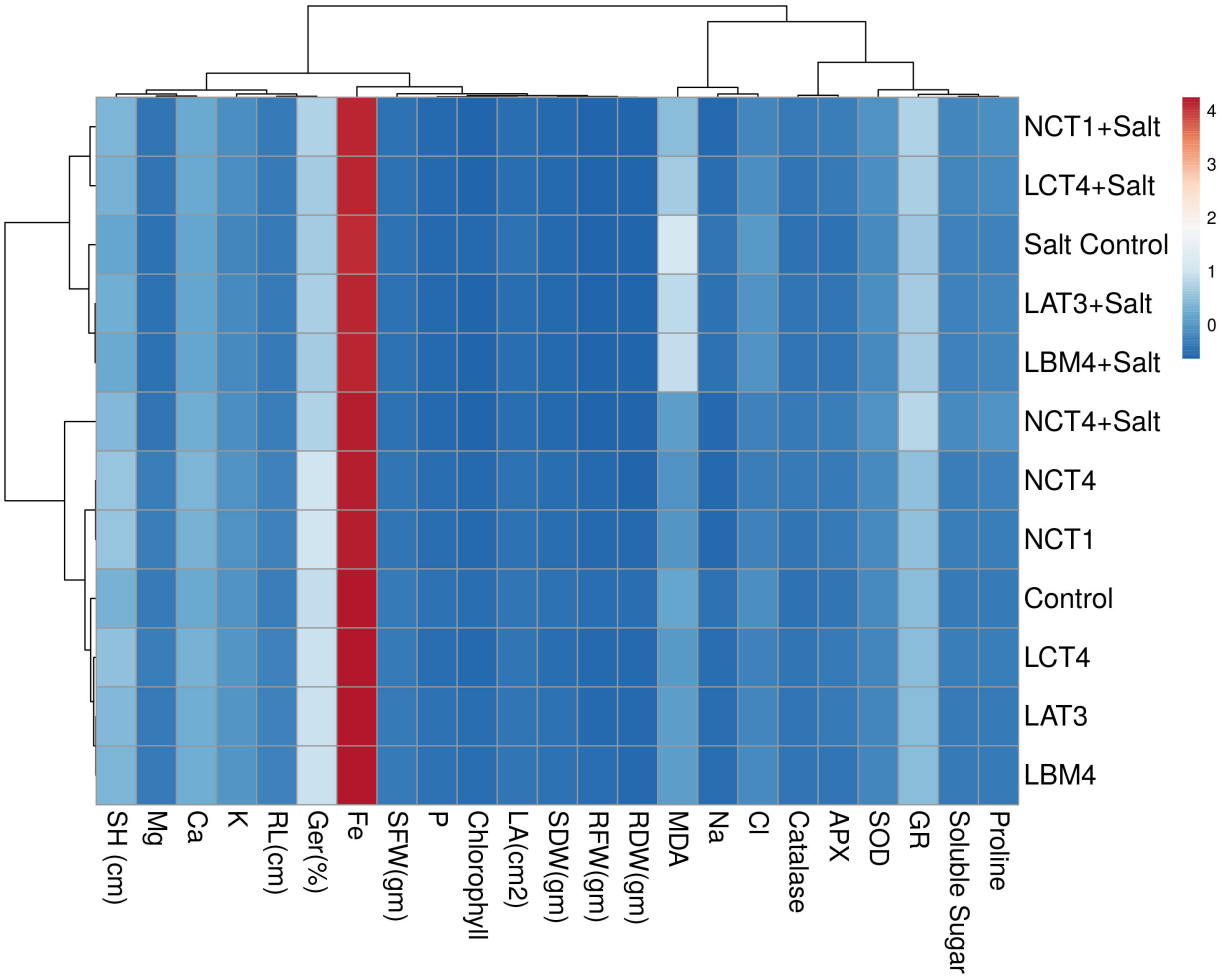
Heatmap represents grouping of control and treated plants in PCA on the basis of growth and physico-chemical parameters.

Similar clusters were also formed by non-metric multidimensional scaling (MDS) (**Figure S1**) which supported the results of multivariate cluster analysis. From the principal component analysis, it was observed that there were 99.99% total variations retained on the basis of the eigen value. The principal component analysis divided the data in to twelve groups on the basis of significant variation in growth and physico-chemical parameters. Group 1 included control plants that varied from treated plants at 98.85% whereas treated plants varied from each other in range from 0.9 to 0.00003% (**Table S2**). The heatmap of principle component analysis also had the similar clustering as observed in multivariate cluster analysis which supports the results (**Figure 7**).

## 4 Discussion

This work successfully established the salt-tolerance and plant growth-promoting abilities of particular PGPR isolates, as well as their impact on enhancing the salinity reduction of tomato S-22 in a greenhouse environment. One essential plant growth-promoting characteristic of PGPR is the synthesis of indole-3-acetic acid, a signal molecule in the control of plant development. In the current investigation, NCT1 produced the most IAA, NCT4 solubilized the most P, and LCT4 produced the most siderophore and ACC deaminase. Additionally, according to Aslam and Ali (2018), ACC-deaminase activity in halotolerant in saline conditions, the bacterial species *Arthrobacter, Brevibacterium, Bacillus, Gracilibacillus, Virgibacillus, Salinicoccus*, *Pseudomonas*, and *Exiguobacterium* encouraged the growth of maize. The remarkable capacity of *B. tequilensis* SSB07 to create biologically active metabolites including gibberellins, indole-3-acetic acid, and abscisic acid was previously demonstrated by Kang et al. (2019). Similar to this, a *Bacillus aryabhattai* (AB211) strain was identified by Bhattacharyya et al. (2017) that produces a clear zone on a Pikovskaya’s agar plate, showing P solubilization. Chookietwattana and Maneewan (2012) previously found *Bacillus megaterium* A12 as a productive halotolerant P solubilizing bacterium in a saline environment. Patel et al. (2023) also found that endophytic *Bacillus safensis* (BS) and rhizospheric *Bacillus haynesii* (BH) strains were able to produce indole-3-acetic acid, gibberellic acid, hydrogen cyanide, ammonia, exopolysaccharides, protease, chitinase, amylase, cellulase, 1-amino cyclopropane-1-carboxylic acid deaminase, and solubilized minerals such as phosphorous, zinc, and potassium. Dutta et al. (2023) reported that *Bacillus* sp. strain PnD, which was isolated from the Indian Sundarban Mangrove Forest, conferred plant growth promoting (PGP) traits like indole 3-acetic acid (IAA) production, phosphate solubilization, and siderophore production.

Additionally, prior studies have demonstrated that PGPR causes the release of metal chelating compounds into the rhizosphere, such as iron chelating siderophores, and that bacteria that produce siderophores have an impact on the uptake of several metals by plants, including Fe, Zn, and Cu. The five PGPR strains utilized in this work can all create siderophore, which is interesting because Dimkpa et al. (2008) discovered that PGPR and other microbes can affect plant stress tolerance by affecting the bioavailability of metal ions needed by their host plants. Lower ethylene levels brought on by the presence of the chosen PGPR containing ACC deaminase generating efficiency could be the cause of the root elongation. Longer roots may have formed as a result of these rhizobacterial strains’ ability to lower endogenous ethylene levels through the activity of ACC deaminase. The additional benefit of this is an increase in shoot height. Under 6 dsm^-1^NaCl stress in the presence of PGPR, Nadeem et al. (2006) noted a comparable elongation in the root length and shoot height of maize. In this investigation, the NCT4, NCT1, and LCT4 strains could produce ACC deaminase (**Table 1**).

Significant changes were found in plant physiological parameters of tomato plants that were affected by PGPR, including germination %, shoot height, root length, leaf area, shoot fresh weight, shoot dry weight, root fresh weight, and root dry weight (**Tables 2**, **3**). According to Jha and Subramanian (2013), paddy rice (*Oryza sativa L.*) ‘GJ-17’ grown in a greenhouse showed 16% greater germination, 27% higher dry weight, and 31% higher plant height in PGPR infected plants under saline conditions. Sen and Chandrasekhar (2014) found that rice genotype ADT43 plants treated with *Pseudomonas* strain greatly enhanced plant height, root length, and dry weight of shoot and root even under salt stress, but plants planted without any treatment grew less. Pérez-Rodriguez et al. (2022) found that *Enterobacter* 64S1 and *Pseudomonas* 42P4 inoculation increased root and shoot dry weight, stem diameter, plant height, and leaf area of tomato compared to control noninoculated plants under saline stress conditions, reversing the effects of salinity. Fan et al. (2016) reported that seed germination, seedling length, vigour index, and plant fresh and dry weight were all increased by inoculating salt-stressed plants with *Arthrobacter* and *Bacillus megaterium* strains. Masmoudi et al. (2021a) also found that *Bacillus velezensis* FMH2-treatment promoted tomato plant growth (root structure, plant elongation, leaf emission, fresh and dry weights, and water content) in absence as well as in presence of salt stress. According to Tank and Saraf (2010), pot experiments on tomato plants stressed with 2% NaCl showed that C4 and T15 were the most successful growth enhancers. When compared to NaCl added untreated seedlings as well as in the absence of NaCl, C4 demonstrated a 50% increase in root length and shoot height. Tomato plants treated with *Enterobacter hormaechei* (MF957335) in saline circumstances dramatically increased their fresh biomass, shoot length, and root length (Ranawat et al., 2021). According to Manh Tuong et al. (2022), under salt stress conditions, the fresh weight of *Stenotrophomonas* sp. SRS1-inoculated *Arabidopsis* and tomato plants was noticeably higher than that of non-inoculated plants. According to Aini et al. (2021), applying saline-tolerant bacteria four times significantly increased plant height (23.36%), leaf area (96.49%), dry weight of the plant (103.59%), and fresh weight of the fruit (85.51%) as compared to not applying bacteria.

In the current study, we found that the levels of both proline and soluble sugar were raised in the PGPR treated tomato plants under salty circumstances. Increased soluble sugar content is another important defense strategy for plants experiencing salt stress (Upadhyay et al., 2012) (**Figure 2**). Proline promotes osmotic adjustment at the cellular level, shielding intracellular macromolecules from dehydration, and it also serves as a hydroxyl radical scavenger, so plants acquire proline as an adaptive response to both general stress and salt. Therefore, through enhancing metabolic defense mechanisms, the PGPR strains most likely aid in promoting plant development under salinity stress (Weisany et al., 2012).

Han and Lee (2005) claim that PGPR, including *Serratia* and *Rhizobium* species, enhance lettuce growth, nitrogen uptake, and chlorophyll content at various soil salinity levels. The most recent research also demonstrates that PGPR inoculation boosted the chlorophyll content of tomato leaves (**Figure 2**). Similar results were obtained by Hahm et al. (2017), who discovered that all three PGPR strains (*Microbacterium oleivorans* KNUC7074, *Brevibacterium iodinum* KNUC7183, and *Rhizobium massiliae* KNUC7586) when inoculated into plants led to higher chlorophyll concentrations than plants that weren’t inoculated.

El-Beltagi et al. (2022) discovered that cherry tomato plants treated with *Azospirillum* and *Azotobacter* had higher leaf chlorophyll content than untreated plants under saline conditions. Inoculating paddy plants with a single PGPR reduced lipid peroxidation by one time, whereas combining two PGPR lowered the level by 1.6 times, according to Jha and Subramanian (2014) research. In both saline and non-saline circumstances, the current study found that PGPR dramatically decreased lipid peroxidation in tomato plants (**Figure 2**). According to Hashem et al. (2016), MDA was negatively impacted by salinity and dramatically elevated by 234.6% in comparison to the *Acacia gerrardii* Benth-saline control. However, *Bacillus subtilis* (BERA 71) inoculation lessened the negative effects of salt on MDA.

Reactive oxygen species (ROS) that are produced as a result of salt stress can injure plants by producing oxidative stress. Superoxide dismutase (SOD), dehydroascorbate reductase (DHAR), catalase (CAT), glutathione reductase (GR), ascorbate peroxidase (APX), and guaiacol peroxidase (GPX) are just a few of the antioxidant enzymes that are involved in antioxidant systems, which are essential for protecting both plants and animals from oxidative stress (Caverzan et al., 2012).

According to El-Esawi et al. (2019) research, plants inoculated with *Azospirillum lipoferum* FK1 shown greater antioxidant gene expression, which boosted the production of antioxidant enzymes and promoted chickpea growth and development. In plant systems, enzymes and redox metabolites cooperate to detoxify ROS. For instance, CAT catalyses the conversion of H_2_O_2_ to oxygen and water, while APX and GPX both catalyze the conversion of H_2_O_2_ to water. No matter the growing conditions (normal or saline), the antioxidant enzyme activities (SOD, CAT, APX, and GR) in leaf extracts of PGPR treated tomato plants were significantly higher than those seen in non-inoculated control plants (**Figure 3**). Our results corroborate those of Gururani et al. (2013), who discovered that PGPR-treated potato plants exposed to various stressors had increased activity of ROS-scavenging enzymes like APX, CAT, DHAR, GR, and SOD (salt, drought, and heavy metals). Khan et al. (2023) also found that antioxidative enzymes (SOD, CAT, APX and GR) increased by 58.40, 25.65, 81.081 and 55.914%, respectively, over salt-treated plants through the application of *Pseudomonas fluorescens*. Additionally, salt-stressed okra plants treated with the PGPR *Enterobacter* sp. UPMR18 showed enhanced SOD, APX, and CAT activities (Habib et al., 2016).

In the current study, it was discovered that the sodium and chloride contents of tomato plants as affected by PGPR in saline and non-saline circumstances were significantly lower (**Figure 4**). Mohamed and Gomaa (2012) found that salinity stress greatly raised the Na^+^ and Cl^-^ concentration in the roots and leaves of radish plants. Inoculating radish seeds with *Pseudomonas fluorescens* and *Bacillus subtilis* greatly lowered the amounts of Na^+^ and Cl^-^. Additionally, Yildirim et al. (2008) found that radish plants inoculated with bacteria under saline stress have lower Na^+^ and Cl^-^ contents than uninoculated plants.

Under both saline and non-saline environments, the study discovered a considerable rise in the magnesium, calcium, potassium, and phosphorus content of tomato plants as impacted by PGPR (**Figure 4**). These results concur with those of Mohamed and Gomaa (2012), who found that the Mg^2+^, Ca^2+^, K, and P contents of radish roots and leaves were decreased by saline stress. Radish seeds were inoculated with *Pseudomonas fluorescens* and *Bacillus subtilis*, which dramatically raised the amounts of Mg^2+^, Ca^2+^, K, and P. Masmoudi et al. (2021b) also reported that *Bacillus spizizenii* FMH45 inoculation significantly decreased endogenous Na^+^ accumulation, increased K^+^ and Ca^2+^ uptake of tomato plants exposed to salt stress. Karlidag et al. (2013) discovered that PGPR inoculation enhanced the Mg^2+^, Ca^2+^, K, and P content of strawberry leaf and root in comparison to non-inoculated plants in salinity conditions. Solórzano-Acosta et al. (2023) found that Rhizobacteria *Pseudomonas plecoglossicida*, and *Bacillus subtilis* contributed to the accumulation of potassium in the leaves, compared to the uninoculated control under salt stress condition.

In the current study, significant variations in tomato plants’ iron content as impacted by PGPR under saline and non-saline conditions were found (**Figure 4**). According to Karlidag et al. (2013), in salinity conditions, PGPR inoculations increased the Fe content of strawberry leaf and root compared to non-inoculated plants. *Kocuria erythromyxa* EY43 had the greatest Fe content, followed by *Bacillus atrophaeus* EY6, *Staphylococcus kloosii* EY37, and *Bacillus spharicus* EY30. Ekinci et al. (2014) claimed that PGPR raised the Fe content of cauliflower.

## 5 Conclusion

According to the current study, *Bacillus* sp., a bacteria that promotes plant growth, can enhance salt stress-induced plant growth and development by solubilizing phosphate and generating ACC deaminase, siderophore, and IAA. Increased levels of ROS-scavenging enzymes (SOD, CAT, APX, and GR), as well as proline and soluble sugar accumulation, which serve as osmoregulants, were also linked to tomato seedlings’ tolerance to salt stress. The goal of this study is to find effective strains that enhance tomato development in both non-stressed and salt-stressed environments. These strains were isolated from the rhizosphere of ‘Kesudo’, ‘Kawaria’, and ‘Arjun’ plants. Accordingly, the results of the present study imply that PGPR can reduce the harmful effects of salt stress on plants, presumably by serving as elicitors that boost plant tolerance to a variety of abiotic stresses.

## 6 Future research

Future research would focus on identifying gene expression and mRNA expression patterns associated with tomatotolerance mechanisms.

## Data availability statement

The original contributions presented in the study are included in the article/**Supplementary Materials**. Further inquiries can be directed to the corresponding authors.

## Author contributions

Conceptualization and supervision: AsP, SB and MJ. Investigation and methodology: AnP, VY and DP. Original draft preparation and analysis: DA, HK and JT. Review and final editing: AsP, MJ, SB and JT. All authors contributed to the article and approved the submitted version.
