# Supplementary figures and images for: Evaluation of the growth-inducing efficacy of various Bacillus species on the salt-stressed tomato (Lycopersicon esculentum Mill.)
Source: Front Plant Sci. 2023 Mar 28;14:1168155. doi: 10.3389/fpls.2023.1168155 (PMC10089305; doi:10.3389/fpls.2023.1168155)

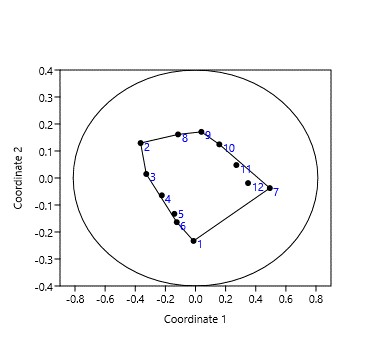

Supplement: Supplementary file 1 [file Image_1.jpeg]
