# Supplementary material for: Evaluation of the growth-inducing efficacy of various Bacillus species on the salt-stressed tomato (Lycopersicon esculentum Mill.)
Source: Front Plant Sci. 2023 Mar 28;14:1168155. doi: 10.3389/fpls.2023.1168155 (PMC10089305; doi:10.3389/fpls.2023.1168155)
Supplement: Supplementary file 2 [file Table_1.docx]

**Supplementary Captions (Figure and Tables)**

**Supplementary Figure 1.** The non-metric multi-dimensional scaling of control and treated plants on the basis of growth and physico-chemical characteristics.

**Supplementary Table 1.** Supplementary Table 1. Correlation analysis of growth and physico-chemical parameters of control and treated plants. Yellow highlights indicate a positive correlation between the parameters, and red highlights indicate a negative correlation.

**Supplementary Table 2.** Principal component analysis of growth and physico-chemical parameters of control and treated plants
