# Supplementary material for: Evaluation of the growth-inducing efficacy of various Bacillus species on the salt-stressed tomato (Lycopersicon esculentum Mill.)
Source: Front Plant Sci. 2023 Mar 28;14:1168155. doi: 10.3389/fpls.2023.1168155 (PMC10089305; doi:10.3389/fpls.2023.1168155)
Supplement: Supplementary file 3 [file Table_2.docx]

**Supplementary Table 1.** Correlation analysis of growth and physico-chemical parameters of control and treated plants

|  | **Soluble Sugar** | **Proline** | **Chlorophyll** | **MDA** | **SOD** | **Catalase** | **APX** | **GR** | **Na** | **Cl** | **Mg** | **Ca** | **P** | **Fe** | **K** | **SH (cm)** | **RL(cm)** | **LA(cm2)** | **Ger(%)** | **SFW(gm)** | **SDW(gm)** | **RFW(gm)** | **RDW(gm)** |
| --- | --- | --- | --- | --- | --- | --- | --- | --- | --- | --- | --- | --- | --- | --- | --- | --- | --- | --- | --- | --- | --- | --- | --- |
| **Soluble Sugar** | 1 |  |  |  |  |  |  |  |  |  |  |  |  |  |  |  |  |  |  |  |  |  |  |
| **Proline** | 0.99211 | 1 |  |  |  |  |  |  |  |  |  |  |  |  |  |  |  |  |  |  |  |  |  |
| **Chlorophyll** | 0.14298 | 0.124384986 | 1 |  |  |  |  |  |  |  |  |  |  |  |  |  |  |  |  |  |  |  |  |
| **MDA** | 0.40428 | 0.374213142 | -0.648911322 | 1 |  |  |  |  |  |  |  |  |  |  |  |  |  |  |  |  |  |  |  |
| **SOD** | 0.92663 | 0.9527337 | 0.307942787 | 0.11055 | 1 |  |  |  |  |  |  |  |  |  |  |  |  |  |  |  |  |  |  |
| **Catalase** | 0.85809 | 0.865998307 | 0.487998553 | -0.07809 | 0.933287 | 1 |  |  |  |  |  |  |  |  |  |  |  |  |  |  |  |  |  |
| **APX** | 0.80156 | 0.812846251 | 0.586752849 | -0.2043 | 0.918388 | 0.984 | 1 |  |  |  |  |  |  |  |  |  |  |  |  |  |  |  |  |
| **GR** | 0.97016 | 0.987884478 | 0.158990562 | 0.27054 | 0.975211 | 0.902 | 0.85 | 1 |  |  |  |  |  |  |  |  |  |  |  |  |  |  |  |
| **Na** | 0.33624 | 0.298159861 | -0.611829171 | 0.98297 | 0.020127 | -0.16 | -0.3 | 0.18 | 1 |  |  |  |  |  |  |  |  |  |  |  |  |  |  |
| **Cl** | 0.25696 | 0.235051846 | -0.743801532 | 0.96992 | -0.03195 | -0.25 | -0.4 | 0.13 | 0.974 | 1 |  |  |  |  |  |  |  |  |  |  |  |  |  |
| **Mg** | 0.29797 | 0.281041609 | 0.951896061 | -0.63698 | 0.463584 | 0.671 | 0.75 | 0.33 | -0.64 | -0.8 | 1 |  |  |  |  |  |  |  |  |  |  |  |  |
| **Ca** | 0.10537 | 0.095019803 | 0.962663576 | -0.75839 | 0.306908 | 0.529 | 0.63 | 0.15 | -0.74 | -0.9 | 0.975 | 1 |  |  |  |  |  |  |  |  |  |  |  |
| **P** | 0.21776 | 0.208806423 | 0.974874583 | -0.64572 | 0.403108 | 0.601 | 0.68 | 0.26 | -0.64 | -0.8 | 0.971 | 0.975 | 1 |  |  |  |  |  |  |  |  |  |  |
| **Fe** | -0.2571 | -0.24382282 | 0.835761065 | -0.95489 | 0.014533 | 0.23 | 0.35 | -0.2 | -0.93 | -1 | 0.812 | 0.907 | 0.834 | 1 |  |  |  |  |  |  |  |  |  |
| **K** | -0.0653 | -0.05993378 | 0.835778574 | -0.88095 | 0.187587 | 0.435 | 0.53 | 0.03 | -0.89 | -1 | 0.884 | 0.941 | 0.88 | 0.955058 | 1 |  |  |  |  |  |  |  |  |
| **SH (cm)** | 0.11707 | 0.111083339 | 0.940057232 | -0.77755 | 0.333377 | 0.558 | 0.65 | 0.18 | -0.78 | -0.9 | 0.975 | 0.993 | 0.964 | 0.912549 | 0.96293 | 1 |  |  |  |  |  |  |  |
| **RL(cm)** | 0.14648 | 0.144323733 | 0.957693475 | -0.76263 | 0.365472 | 0.572 | 0.67 | 0.21 | -0.76 | -0.9 | 0.976 | 0.993 | 0.98 | 0.90578 | 0.94066 | 0.99212 | 1 |  |  |  |  |  |  |
| **LA(cm2)** | -0.1548 | -0.15305312 | 0.910436232 | -0.89145 | 0.090819 | 0.302 | 0.42 | -0.1 | -0.87 | -0.9 | 0.881 | 0.955 | 0.898 | 0.980818 | 0.95103 | 0.95061 | 0.94577 | 1 |  |  |  |  |  |
| **Ger(%)** | -0.342 | -0.34172351 | 0.842062203 | -0.91793 | -0.09736 | 0.123 | 0.25 | -0.3 | -0.88 | -0.9 | 0.782 | 0.892 | 0.818 | 0.981474 | 0.92249 | 0.88682 | 0.87382 | 0.9756 | 1 |  |  |  |  |
| **SFW(gm)** | -0.2567 | -0.24005001 | 0.707725853 | -0.88103 | 0.008031 | 0.248 | 0.32 | -0.1 | -0.89 | -1 | 0.726 | 0.806 | 0.754 | 0.917744 | 0.9272 | 0.84498 | 0.81544 | 0.8658 | 0.8925021 | 1 |  |  |  |
| **SDW(gm)** | -0.251 | -0.23386698 | 0.713042885 | -0.88197 | 0.015171 | 0.253 | 0.33 | -0.1 | -0.9 | -1 | 0.731 | 0.809 | 0.758 | 0.919607 | 0.92846 | 0.84858 | 0.81933 | 0.8693 | 0.8935199 | 0.999825 | 1 |  |  |
| **RFW(gm)** | 0.06328 | 0.070111511 | 0.957875204 | -0.80091 | 0.298223 | 0.483 | 0.59 | 0.14 | -0.78 | -0.9 | 0.936 | 0.975 | 0.962 | 0.927607 | 0.91491 | 0.9636 | 0.98424 | 0.9617 | 0.8965124 | 0.798944 | 0.8034515 | 1 |  |
| **RDW(gm)** | 0.03979 | 0.051042122 | 0.953244357 | -0.80763 | 0.283968 | 0.462 | 0.57 | 0.12 | -0.78 | -0.9 | 0.925 | 0.967 | 0.955 | 0.931919 | 0.90932 | 0.95648 | 0.97732 | 0.9624 | 0.9021303 | 0.8113854 | 0.8161694 | 0.99753766 | 1 |
