# Supplementary material for: Evaluation of the growth-inducing efficacy of various Bacillus species on the salt-stressed tomato (Lycopersicon esculentum Mill.)
Source: Front Plant Sci. 2023 Mar 28;14:1168155. doi: 10.3389/fpls.2023.1168155 (PMC10089305; doi:10.3389/fpls.2023.1168155)
Supplement: Supplementary file 4 [file Table_3.docx]

**Supplementary Table 2.** Principal component analysis of growth and physico-chemical parameters of control and treated plants

| Principle component analysis | | |
| --- | --- | --- |
| PC | Eigenvalue | % Variance |
| 1 | 35067.5 | 98.852 |
| 2 | 344.064 | 0.96988 |
| 3 | 52.518 | 0.14804 |
| 4 | 8.83942 | 0.024918 |
| 5 | 0.951953 | 0.002684 |
| 6 | 0.482998 | 0.001362 |
| 7 | 0.130845 | 0.000369 |
| 8 | 0.110113 | 0.00031 |
| 9 | 0.074511 | 0.00021 |
| 10 | 0.037809 | 0.000107 |
| 11 | 0.019449 | 5.48E-05 |
| 12 | 0.010831 | 3.05E-05 |
|  |  | 99.99996 |
